# Production of rhamnolipids by integrated foam adsorption in a bioreactor system

**DOI:** 10.1186/s13568-018-0651-y

**Published:** 2018-07-24

**Authors:** Iva Anic, Ines Apolonia, Pedro Franco, Rolf Wichmann

**Affiliations:** 1 Laboratory of Biochemical Engineering, Department of Biochemical and Chemical Engineering, TU Dortmund University, Emil-Figge Straße 66, 44227 Dortmund, Germany; 2 Department of Bioengineering, Instituto Superior Técnico, University of Lisbon, Av. Rovisco Pais 1, 1049-001 Lisbon, Portugal

**Keywords:** Biosurfactant, Foam fractionation, Foam adsorption, Rhamnolipids, Integrated process, Process intensification

## Abstract

Biosurfactants offer environmental as well as health benefits over traditionally used chemical surfactants and heterologous production from engineered microorganisms has been demonstrated, offering containable as well as scalable production of these alternative chemicals. Low product titers and cost intensive downstream processing are the main hurdles for economical biosurfactant production at industrial scales. Increased biosurfactant concentrations are found in the liquid fraction of the foam formed during fermentation of producing microbes. Adsorption of biosurfactants from foam fractions in cultivations may offer a simple concentration and purification method which could enable their cost-effective production. Here, foam adsorption was applied as an in situ method for separation of the rhamnolipid biosurfactants during fermentation of *Pseudomonas putida* EM383. An integrated process was designed to capture the produced rhamnolipids on hydrophobic adsorbent in packed bed units while minimizing the impact of adsorption on the productivity of the system by recirculating cell-containing collapsed foam flow-through back into the reactor vessel. A stable rhamnolipid production by *P. putida* EM383 on glucose was performed coupled to this adsorption strategy for 82 h, after which no remaining rhamnolipids were found in the cultivation broth and 15.5 g of rhamnolipids could be eluted from the adsorbent. Rhamnolipid yield from glucose feed was 0.05 g g^−1^, when up to 2 g L^−1^ glucose pulse feeding was applied. After solvent evaporation, a product purity of 96% was obtained. The results indicate that the integrated adsorption method can be efficient for simultaneous production and recovery of rhamnolipid biosurfactants from microbial fermentations.

**Electronic supplementary material:**

The online version of this article (10.1186/s13568-018-0651-y) contains supplementary material, which is available to authorized users.

## Introduction

Biosurfactants are thought to be efficient alternatives and possible enhancers of chemically synthesized surface active agents, as they are in comparison biodegradable, less toxic, more effective at extreme temperature and pH values, and can be produced from renewable resources (De et al. 2015). The relative importance of surfactants and biosurfactants today is indicated by the size of the markets for these materials and their market growth rate (Chandra 2015). One of the most extensively studied biosurfactants in terms of their industrial and environmental application are the rhamnolipids due to their potential applications in a wide variety of industries and the high production titers by the pathogenic bacterium *Pseudomonas aeruginosa*. Large-scale rhamnolipid production is hindered by the intrinsic health hazard of cultivating *P. aeruginosa* (Toribio et al. 2010), which can produce up to 39 g L^−1^ rhamnolipids (Müller et al. 2010). Although two other non-pathogenic bacteria also produce rhamnolipids, *Burkholderia thailandensis* natively at the titer of 1.5 g L^−1^ (Dubeau et al. 2009) and *Pseudomonas putida* only heterologously, the producing titers from these organisms are insufficient to warrant industrial scale processes. Production costs of biosurfactants also prevent them from competing with their synthetic counterparts (Kaskatepe and Yildiz 2016). Optimization of growth and production conditions using renewable and low-cost substrates as well as the development of efficient multi-step downstream processing will be necessary to produce biosurfactants in more economically feasible ways (Mulligan et al. 2014). Conventional technologies used for purification of rhamnolipids and process time requirements are significant cost-factors which can account for a large proportion of the total production costs (Banat et al. 2014). High production costs are mainly due to the need for preventative control of intense foaming, complex downstream process and use of expensive substrates for the production.

The most common practice in the foam destruction is the use of antifoam chemicals and mechanical foam breaking devices. However, these steps add to the complexity as well as cost of the downstream process and are insufficient for foam destruction in vigorously foaming biosurfactant systems (Winterburn and Martin 2012). Research efforts regarding biosurfactant production processes have been directed towards development of more effective downstream processes. For this reason, there is an interest in utilizing controlled foaming in biosurfactant fermentation systems through the application of foam separation techniques (Chen et al. 2006a). One of the emerging technologies applied for this purpose leverages the native partitioning of surface active compounds into foam fractions during fermentation processes and is termed the foam fractionation method. Foam fractionation is a process whereby dissolved or colloidal material is selectively adsorbed on the surface of rising bubbles and then is partially segregated by the foam (Lemlich 1972).

As biosurfactants are naturally surface-active substances, during the cultivation they partition into the foam and are concentrated in it. Resulting biosurfactant-rich foam can be captured as a separate phase. Several reports have described implementation of foam fractionation for biosurfactant concentration. Integrated foam fractionation was reported in processes for production of rhamnolipids (Heyd et al. 2011; Beuker et al. 2016), surfactin (Chen et al. 2006a; Alonso and Martin 2016), hydrophobin protein HFBII (Winterburn et al. 2011), and mycosubtilin (Guez et al. 2007). In these processes, foam fractionation and product separation were conducted by either using a fractionation column attached directly to the headplate of the bioreactor or by allowing foaming inside the bioreactor space. Rhamnolipid enrichments factors of 4 (Sarachat et al. 2010), 15 (Beuker et al. 2016) and 53 (Heyd et al. 2011) in the foam phase were reported, with product recovery in the foam phase as high as 97% (Sarachat et al. 2010; Beuker et al. 2016). The high degree of biosurfactant concentration in the foam fraction can save incredible energy input requirements in downstream purification processes (Stevenson and Li 2014).

Nevertheless, an important drawback of foam fractionation is simultaneous cell accumulation in the foam, which leads to a continuous reduction of biomass concentration in the fermentation broth (Kosaric and Vardar-Sukan 2015). A simple foam fractionation method can be applied in cases when the biomass enrichment in the foam is low, but there are only few examples where low biomass enrichments in the foam are described (Beuker et al. 2016), otherwise the loss of the producing microorganism has a significant influence on production efficiency (Davis et al. 2001). Foam affinity for various cells is different (Shedlovsky 1948) and it is not yet clear which properties and system variables influence the cell attachment to foam bubbles. Strain selection, genetic engineering and medium modification were suggested to reduce cell hydrophobicity and address the foaming issue of rhamnolipid fermentation (Sodagari and Ju 2014). A subsequent problem of foam is that conventional exponential feeding strategies indeed fail to account for the loss of biomass caused by foaming (Chenikher et al. 2010). The volume of foam created in the process needs to be accounted for as well, due to the resulting loss of nutrients and liquid volume that make up the liquid fraction of the foam. Cultivation with integrated foam fractionation and separation is not feasible as long as no cell retention method is employed. This generates a necessity for implementation of some kind of cell retention method, such as cell immobilization in magnetic alginate beads (Heyd et al. 2011). Product separation and subsequent cell recirculation into the bioreactor were also suggested (Winterburn et al. 2011).

Recently, we described a novel separation technology for rhamnolipid recovery from fermentative processes wherein rhamnolipid rich, cell-containing foam was directed onto an adsorbent, resulting in rhamnolipid depletion and consequent foam collapse (Anic et al. 2017). Selection of an appropriate adsorbent material with large enough spherical particles ensured sufficient void space for air, cells and nutrient broth to flow through a fixed bed of the adsorbent. In this study, an efficient implementation of this in situ separation technique in bioprocess production is presented. Specific variables of the integrated process are discussed and compared with reported processes for production of biosurfactants using integrated foam fractionation.

## Materials and methods

### Chemicals

All chemicals were purchased from Carl Roth GmbH, (Karlsruhe, Germany) if not stated otherwise. All of chemicals and reagents were analytical grade.

### Rhamnolipid producing microorganism

The non-pathogenic, mono- and di-rhamnolipid producing *P. putida* EM383 which carries plasmid pPS05_rhlAB described in Tiso (2016) was used in this study. This strain was kindly provided from Lars Blank, Institute of Applied Microbiology, RWTH Aachen University, Germany. Fermentations were performed with modified Riesenberg medium, as previously described (Anic et al. 2017). The pH of medium was corrected to pH 6.8 using 6.5 M NH_4_OH solution and further to 7 with 10 M NaOH solution. Tetracycline was added to a final concentration of 20 mg L^−1^ in medium. Glucose was used as a sole carbon source in all fermentations. The feeding solution contained 700 g L^−1^ glucose, 22 g L^−1^ KH_2_PO_4_, 0.01 g L^−1^ FeSO_4_∙7H_2_O and 0.1∙10^−3^ g L^−1^ CuCl_2_∙2H_2_O.

### Cultivation conditions, integrated foam capture, and bioreactor set-up

Each of three 1 L shake flasks filled with 200 mL of fermentation medium containing 5 g L^−1^ glucose was inoculated with 300 µL of glycerol stock solution of *P. putida* EM383. The pre-culture was incubated in a cell incubator shaker at 33 °C and 200 rpm at 25 mm throw diameter for 13 h when it reached the mid-exponential phase. All of the 600 mL were used to seed bioreactor fermentations to the starting biomass concentration of 0.1 g L^−1^. The 3.1 L bioreactor (KLF 2000, Bioengineering, Switzerland) was equipped with an integrated pH, temperature and aeration control system. During bioreactor cultivation, aeration was set to 0.1 vvm and pO_2_ was controlled at 20% via stirring rate. Stirring was provided with two blade-stirrers on the motor drive shaft and the stirring rate was regulated by the signal of the pO_2_. The stirring range was 300–1200 rpm. The temperature was held constant at 33 °C while the pH was controlled and automatically adjusted to 7.0 ± 0.5 by addition of 10 M NaOH or 3 M HCl. Initial glucose concentration of 14 g L^−1^ in the bioreactor was set by addition of 50 mL of sterile glucose feeding solution. Fermentation medium was added to a final working volume of 2.5 L. During the fed-batch phase, that was started 24 h after the fermentation start, glucose concentration in the bioreactor was controlled every 0.5 h and increased to a maximum of 2 g L^−1^ by pulsed addition of feeding solution. Resulting feeding profile (see Additional file 1: Figure S1) was applied in all subsequent fermentations.

An exhaust-gas line on the top of the bioreactor was connected to a foamate container composed of a simple 10 L pressure-stable glass bottle equipped with a sterile filter for fermentation gas exhaust on its top opening and a bottom outlet for broth recirculation. Due to slightly increased pressure in the bioreactor during integrated fermentation, feed and pH correction solutions were injected via the foamate container into the system. The container outlet was connected to the bioreactor needle inlet via tube connection and collapsed foam liquid was pumped back into the bioreactor via an in-line peristaltic pump.

### Cultivation with an in-line integrated adsorption column and product separation

**Fig. 1:**
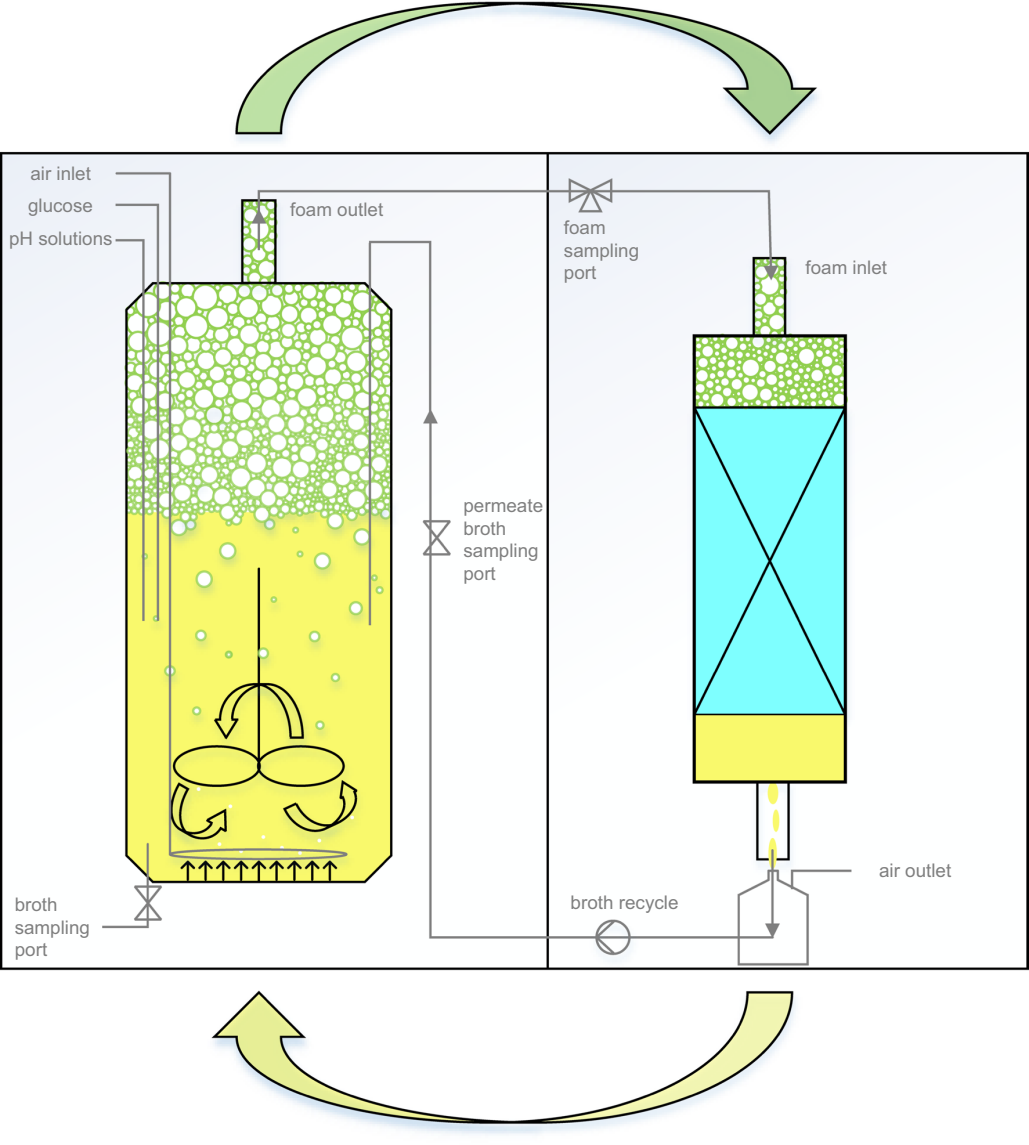
Concept of rhamnolipid separation from fermentation broth using foam technology principle

The setup described above was completed with an automated adsorption unit for rhamnolipid separation. The adsorption unit was installed between the top of the bioreactor and the foamate container. The outlet of the bioreactor off-gas line was connected to the inlet of the adsorption unit. The technology principle for rhamnolipid separation is shown in Fig. 1.

**Fig. 2:**
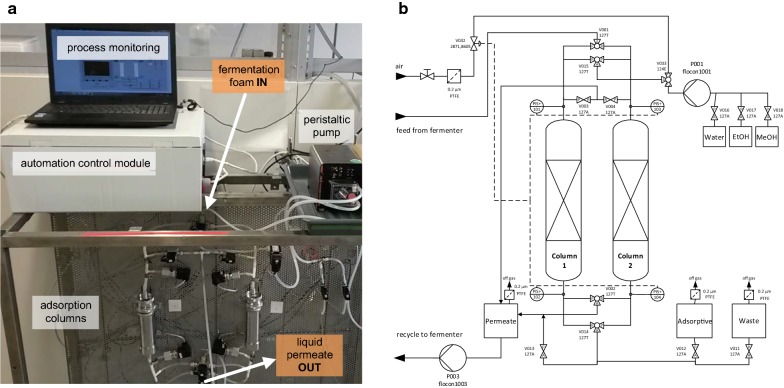
Laboratory setup (**a**) and process flow sheet (**b**) of automated adsorption unit. Adsorption columns were used alternatively, where adsorption phase was set to 12 h. In non-adsorptive column mode, elution and preparation for the next run were performed

The automated adsorption unit consisted of two adsorption columns that can be operated alternatively, as presented in the Fig. 2a. Each of two stainless steel columns consisted of a packed bed section (h = 1.25 cm, d = 3.8 cm) fixed with nylon meshes on the bottom and on the top and an additional upper column head section (h = 7.0 cm, d = 4.2 cm) to ensure uniform foam distribution within the column. Both columns were packed with 5 g of hydrophobic C18 silica-based adsorbent ODS-A (Octadecylsilyl-A AA12SA5 12 nm, S-150 μm; YMC, Japan). Prior to adsorption, columns were wetted with 1 bed volume (BV) of sterile 99.8% (w/v) denatured ethanol to remove any impurities and then washed with 3 BV of sterile bi-distilled water. Finally, sterile air was blown through for 1 min at a flow rate of 0.5 L min^−1^ to dry the columns prior to process operation. All the experiments within integrated and non-integrated system were performed under sterile conditions.

Foam that was created in the fermentation head space was allowed to stream into the adsorption column and liquid permeate which came through the packed bed was pumped back into the bioreactor via the foamate container. The off-gas released from the foam bubbles was separately led off through the air filter on the top of the foamate container. During the automated adsorption process, the pressure drop along the adsorption column was monitored by pressure indicators for the pressure range of 0 up to max. 1.5 bar, which is the highest allowed pressure applicable on the bioreactor vessel used. The columns were operated alternatively and each column was in adsorption mode for 12 h. Elution and column wash was performed in the following order: 3 BV of sterile bi-distilled water, followed by 3 BV of sterile 99.8% ethanol solution, 1 BV of sterile 99.9% methanol solution, and finally 3 BV of sterile bi-distilled water. All the washing steps were performed at a flow rate of 14 mL min^−1^ and between each of the washing steps sterile pressurized air was blown for 1 min at a flow rate of 0.5 L min^−1^, to ensure that no mixing of liquid solutions occurred. Liquids were pumped by peristaltic pump connected to bi-distilled water, ethanol, and methanol containers. After the washing and elution steps, the solutions were collected in fractions. Communication between the valves, pumps and other adsorption unit elements was established via programmable control module according to the scheme presented in Fig. 2b.

## Analytical methods

### Sampling and processing

Biomass, rhamnolipids, and glucose concentrations were measured from samples of fermentation broth or fermentation foam taken aseptically at the bottom of the bioreactor, at the off-gas exhaust as well as behind the adsorption column. For biomass determination, OD_600_ was measured and multiplied by a gravimetrically determined biomass correlation factor of 0.397 to calculate the mass in g L^−1^. Fermentation samples were centrifuged for 5 min at 13,000×*g* and filtered through cellulose acetate filter with a pore size of 0.2 µm. The filtrate was diluted with acetonitrile (J.T. Baker, Phillipsburg, NJ, USA) and was left for 4 h at a temperature of 4 °C. Samples were centrifuged for 3 min at 13,000×*g* and the supernatant was transferred into HPLC vials. Detection of rhamnolipids in samples was performed using a HPLC method as previously described (Tiso et al. 2016; Anic et al. 2017). Glucose concentrations were detected from the aqueous phase of samples using a glucose assay kit (Cat. No. 10 716 251 035, R-Biopharm AG, Germany), according to the manufacturers’ instructions. The resultant rhamnolipid elutions of each fermentation run were pooled separately and transferred to a round-bottom flask connected to a rotary evaporator. The concentration process was continued at 40 °C until a consistently viscous precipitate of crude biosurfactant was obtained, which was then freeze-dried.

### Data analysis

All experimental data, except for fermentations, are presented as arithmetic averages of at least three replicates. Fermentations were performed in duplicates. Standard deviations are indicated by error bars.

Volumetric productivity was calculated as the maximum total rhamnolipid concentration c_RL final_, divided by the time t_production_ to reach that concentration:

1 $${\text{Volumetric productivity (mg L}}^{ - 1} \;{\text{h}}^{ - 1} ) = \frac{{{\text{c}}_{\text{RL final}} }}{{{\text{t}}_{\text{production}} }} \cdot 1 0 0 0$$

Biomass and rhamnolipid yield on glucose was calculated from final concentrations, where mass of total produced cells or rhamnolipids m_produced_ was divided by the mass of consumed glucose m_Glc consumed_:

2 $${\text{Y}}_{\text{of RL or cells on Glc}} = \frac{{{\text{m}}_{\text{cells produced}} }}{{{\text{m}}_{\text{Glc consumed}} }}$$

Overall mass of cells and rhamnolipids produced was calculated as the sum of masses found in the fermentation broth m_in the fermentation broth_, in the collapsed foam m_in the collapsed foam_, and, in integrated system and for rhamnolipids only, of eluted rhamnolipid mass m_eluted_:

3 $${\text{m}}_{\text{produced}} = {\text{(c}} \cdot {\text{V)}}_{\text{in the fermentation broth}} + {\text{(c}} \cdot {\text{V)}}_{\text{in the collapsed foam }} + {\text{(c}} \cdot {\text{V)}}_{\text{eluted}}$$

Bacterial and rhamnolipid enrichment was calculated using bacterial and rhamnolipid concentration values in the foamate and in the bioreactor. The concentration of a component in the foamate c_in the foam_ was divided by its mean concentration in the bioreactor c_in the fermentation broth_ as follows:

4 $${\text{Enrichment}} =\frac{{{\text{c}}_{\text{in the foam}} }}{{{\text{c}}_{\text{in the fermentation broth}} }}$$

Rhamnolipid recovery was calculated from the ratio of eluted mass of rhamnolipids m_RL eluted_ over total produced mass of rhamnolipids present in the fermentation system m_RL produced_, as follows:

5 $${\text{Rhamnolipid recovery}}\, (\%) = \frac{{{\text{m}}_{\text{RL eluted}} }}{{{\text{m}}_{\text{RL produced}} }} \cdot 100$$

For the determination of rhamnolipid purity, freeze dried samples were weighed and diluted in acetonitrile. Samples were taken and rhamnolipid content was determined by HPLC. Product purity was calculated from rhamnolipid mass in the lyophilizate powder m_RL_ and the total mass of the lyophilizate m_total product_ using following equation:

6 $${\text{Rhamnolipid purity }}(\%) = \frac{{{\text{m}}_{\text{RL}} }}{{{\text{m}}_{\text{total product}} }} \cdot 1 0 0$$

## Results

Two fermentation setups were designed and used in this work. A reference set-up was used wherein the foam fraction was recirculated into the reactor without rhamnolipid capture. Simultaneous rhamnolipid separation was performed in a second set-up with a packed bed adsorption column integrated on the top of the bioreactor off-gas line. Average values of the duplicate fermentations are presented.

### Biomass, rhamnolipid, and glucose concentrations during bioreactor cultivations

**Fig. 3:**
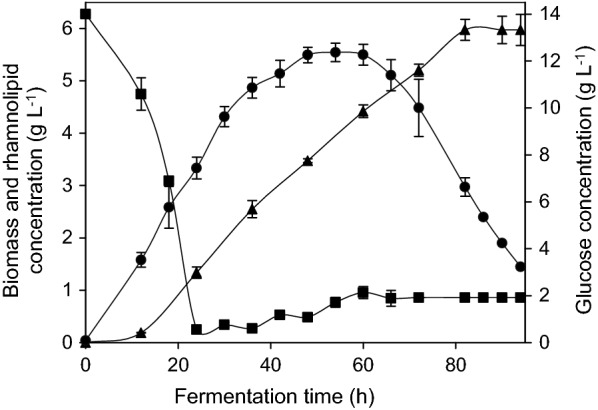
Time course of overall biomass, glucose and rhamnolipid concentrations during cultivations of integrated fermentation are shown. Feed was started at time 24 h. Values of biomass (circles), glucose (squares) and rhamnolipids (triangles) concentrations of two integrated cultivations are given as mean value

Overall biomass, rhamnolipids, and glucose concentrations during fermentations are depicted in Fig. 3.

No differences in biomass growth or overall rhamnolipid production were observed between fermentations with and without integrated foam adsorption and glucose concentrations during the fermentation were similar in both fermentations. When the culture reached the stable log phase after 24 h, and the starting glucose concentration of 14 g L^−1^ was reduced to 0.5 g L^−1^, the fed-batch phase was started by feeding of glucose solution. A maximum biomass concentration of 5.5 g L^−1^ and a maximum rhamnolipid concentration of 6.0 g L^−1^ were reached after 82 h of cultivation in 2.6 L working volume. Rhamnolipid production was observed after 12 h of the cultivation in both fermentation systems, indicating a partially growth-associated rhamnolipid production as already described for heterologous *P. putida* (Wittgens et al. 2011). After 82 h of fermentation, in both systems foam formation ceased and no further rhamnolipid accumulation in the nutrient broth was detected. Further biomass degradation and glucose accumulation was observed in both fermentation systems. Volumetric productivity of 73 mg L^−1^ h^−1^, product yield on glucose of 0.05 g rhamnolipids g^−1^ glucose and biomass yield of 0.046 g cell dry weight g^−1^ glucose were calculated according to Eqs. 1 and  2.

### Foam formation, biomass and rhamnolipid enrichment

Foaming of fermentation broth on the liquid surface started directly at the beginning of fermentation with both setups due to the presence of rhamnolipids in seed cultures. This foam was unstable and consisted of large foam bubbles with high concentrations of interstitial liquid. After 12 h of fermentation, rhamnolipid concentrations increased, foam became drier and compact with smaller bubbles which began to exit the bioreactors. In the reference fermentation, all foam was directed into the foamate container which was used as an extension of the bioreactor head space wherein a longer retention time allowed foam to collapse before recirculation. The foam fraction contains whole culture broth including cells and rhamnolipids which could be continuously pumped back into the bioreactor after being collected in the foamate container. Although recirculation of collapsed foam into the reactor could maintain a steady state, the volume of stable slow-collapsing foam continually increased throughout fermentation and reached volume-space of 10 L. Once collapsed, the measured volume of rhamnolipid-containing solution generated from this foam was 10 mL and biomass and rhamnolipid concentrations were 1.2 times higher than the concentrations measured in the bioreactor liquid. Final rhamnolipid concentration in non-integrated system was calculated to be 5.9 g L^−1^ by addition of rhamnolipid mass present in the fermentation broth inside the bioreactor and in the foamate in the foamate container. The final biomass concentration was calculated in the same manner, as shown in the Eq. 3.

**Fig. 4:**
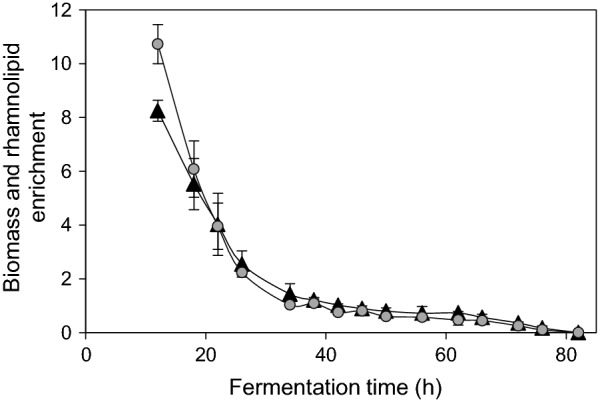
Time course of differential bacterial (circles) and rhamnolipid (triangles) enrichment in the foam during integrated fermentations. Foam samples were taken at the sampling port on the foam exit at the top of the bioreactor. The values for bacterial and rhamnolipid enrichment were calculated according to Eq. 4

Same trend in foam formation was observed in both fermentation setups. However, when foam contacted the adsorbent material, it rapidly collapsed and the resultant liquid cell-containing culture broth, which was found to be highly depleted of rhamnolipids, was collected in the foamate container and could be continuously recirculated in the bioreactor. Residence time of the cells in the adsorption column varied over the fermentation time dependent on the foam flow velocity. A residence time of ~ 1 min in the system outside of the bioreactor before being pumped back together with the surrounding fermentation broth into the fermentation vessel was measured. The cells were transferred by the air flow along with the nutrient broth through the packed adsorption bed for 5–15 s. Biomass and glucose concentrations measured at the entrance were not observed to deviate from the values measured at the exit of the adsorption column for the whole fermentation time. As the adsorption columns were dismounted at the end of the process run, no biofouling of adsorption material was observed. Rhamnolipid concentrations in permeate at the exit of the adsorption column were found to be between 0 and 0.05 g L^−1^. No foam formation in the line after the adsorption column was observed. Similar trends in bacterial and rhamnolipid enrichment were observed in both fermentations as depicted in Fig. 4. Bacterial and rhamnolipid enrichment in the foam was high as 10.7 and 8.3 at the beginning of foam production, respectively, and decreased continually throughout the cultivation to concentrations in the foam 10 times lower than the concentrations in the broth.

**Fig. 5:**
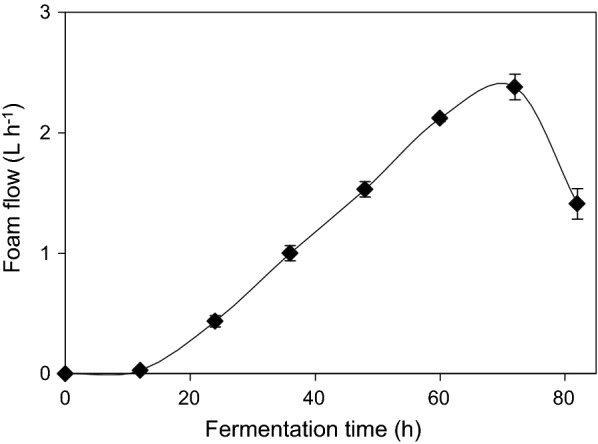
Volumetric flow of the foam during the integrated fermentations. Foam volume and bacterial and rhamnolipid concentrations were measured after 30 min long foam collapse in the collection tube at room temperature

For the first 20 h of foam formation, biomass enrichment in the foam fraction was slightly higher than rhamnolipid enrichment. After 34 h, no increase in cell or rhamnolipid concentration was observed in fermentation foam compared to fermentation broth, although both cells and rhamnolipids were present in the foam. Although foam formation increased over the early stages of cultivation, rhamnolipid enrichment in the foam reduced as foam formation and flow-rates increased, which was coupled to an increased cell concentration in the foam fraction. Foam flow in the range of velocity from 0.5 to 38 mL min^−1^ was observed in both fermentations as depicted in Fig. 5. Towards the end of the fermentation, foam production reduced and finally ceased after which the fermentation runs were ended. Finally, no rhamnolipids were present in the fermentation broth, and the rhamnolipid production lasted for 82 h. This means that all the present rhamnolipids were captured in the adsorption column material, thus recovery from the fermentation broth was 100%.

### Rhamnolipid recovery from adsorbent and further purification

Integrated adsorption of rhamnolipids from fermentation foam allowed the complete recovery of these biosurfactants from adsorbent columns by elution with ethanol and methanol solutions. Two adsorption columns were operated alternatively, each for 12 h of adsorption and subsequent 20 min of product elution and column washing. Adsorption capacity of the adsorbent was reported to be 0.38 g rhamnolipid g^−1^ adsorbent (Anic et al. 2017), meaning that 1.9 g rhamnolipid was the maximum to be adsorbed on each of the adsorption columns in one adsorption cycle. The expected adsorption capacity of packed columns was not exceeded since no foam formation behind the column was observed. Switch of foam stream direction from one column to another was controlled using electrically controllable in-line valves. Eluates from the adsorbent columns were subject to rotary drying, and the resultant rhamnolipids were observed to be a viscous light-yellow–brown product which could be lyophilized to a powder. When all elution fractions were pooled from an individual reactor run, 15.54 ± 0.2 g of rhamnolipids were obtained at 96 ± 1.2% purity consisting of 90% di- and 10% monorhamnolipids. Calculated biomass and rhamnolipid concentrations are the same in both processes during the fermentation and at the fermentation end, as presented in the Eq. 3.

Therefore, it can be concluded that 100% rhamnolipid recovery was conducted using the integrated adsorption column.

## Discussion

### Rhamnolipid production with integrated foam adsorption

In this work, a novel separation method was successfully integrated into fermentative production of rhamnolipids in a bioreactor. A comparison fermentation was performed without rhamnolipid separation where a rhamnolipid containing foamate was allowed to collapse and recirculated into the bioreactor along with cells and nutrients. Results of fermentation without separation are presented alongside results from fermentation with integrated rhamnolipid separation.

#### Foam collapse

During rhamnolipid production in both fermentations, intensive foaming led to a discharge of surface-active rhamnolipids and bacteria from the bioreactor. To maintain bacteria inside the bioreactor in reference fermentations, the discharged foam was collected in a separate large bottle and pumped back into the bioreactor, as previously proposed (Heyd et al. 2011). In the integrated fermentation, foam was allowed to stream through an adsorption column, resulting in an effluent of cell-containing nutrient broth devoid of rhamnolipids. Instant foam collapse occurred due to hydrophobic–hydrophobic interaction (HHI) between the rhamnolipids and the hydrophobic ligands of the surface of the adsorbent, indicating the potential value of this technique for separation of amphiphilic compounds and foam removal in fermentation systems.

#### Cell retention

Foam recovery systems and further recycling of cells from foamate can improve microbial performance in fermentation systems (Alonso and Martin 2016). Most in situ product removal (ISPR) systems have overlooked the contribution of cells separated with the ISPR and their recirculation into the cultivation stage. Cell growth, Y_X/S_ and Y_P/S_ were improved when cells were foamed out and re-used for production of surfactin (Alonso and Martin 2016). Previous studies have shown that the limitation of integrated surfactin production with a foam separation strategy resulted in lower productivities caused by significant carryover of biomass with the foam (Chen et al. 2006b). Rhamnolipid yield could be improved by 83% when foam formation was reduced by applying a stop valve (Long et al. 2017). It has also been shown that a stripping column can reduce biomass loss to 5% over the whole course of the fermentative production of hydrophobin protein HFBII (Winterburn et al. 2011). Higher and larger fractionation columns or multistage foam fractionation was also suggested for cell retention during rhamnolipid production (Heyd et al. 2011).

In both, fermentations with and without integrated foam adsorption, foaming and thus biomass and rhamnolipid carryover occurred. Both systems were operated in a closed loop and in both of them all the cells that were transferred with the foam were recycled back into the bioreactor by pumping: either the permeate liquid flowing from the adsorption column or collapsed foam. Analysis shows no difference in biomass enrichment between the two fermentation setups. Cell biomass loss in the non-integrated system was not significant and this fermentation was used as a representative control-case. Foam collapse occurred within few minute and the cells were robust enough to suffer the anaerobic conditions for short time, which was necessary to transfer the collapsed cell-containing broth by pumping into the bioreactor. A loss of 0.5% was observed in the very stable foam phase which was calculated from a mass balance of the volume of slowly collapsed foam, a bacterial enrichment, and total cell mass produced in the system.

In the integrated production system, cells and glucose do not undergo HHI on an adsorbent with hydrophobic ligands and here, cells were observed to flow through the adsorption column, allowing fast recirculation into the bioreactor to ensure continued productivity. Using an adsorbent with an appropriate particle size and properties, it was possible to separate the rhamnolipids and to permeate the cells back into the bioreactor by simple pumping during the 82 h of fermentation, without the need for cell retention or separation by centrifugation or filtration.

#### Productivity

Volumetric productivity of rhamnolipids in both reference and integrated rhamnolipid adsorption systems was 73 mg L^−1^ h^−1^. Since both fermentations were identical apart from simultaneous rhamnolipid separation, rhamnolipid removal appears to have had no influence on the system productivity. Since the height of the adsorption bed is 1.25 cm, a short retention time of cells in the adsorption column was expected. The rhamnolipid production in both tested systems was the same, so the residence time in the adsorbent bed had no influence on the cell productivity. The cells that were present in the system outside of the bioreactor in non-integrated system did not undergo nutrient-limited conditions as these cells were transferred within a liquid nutrient broth-phase pushed by the aeration in the system.

It is not clear if the residence time in the foam and collapsed foam phase outside of the bioreactor has an influence on the cell productivity. Cell foaming was described to result in 1.13-fold enhanced production of surfactin when coupled to cell recirculation in fermentation with *B. subtilis*. Foaming might have had positive influence on the volumetric productivity of surfactin from *B. subtilis*, although, it is not known if this behavior is strain-specific or a particular feature resulting from the foaming processes (Alonso and Martin 2016).

*Pseudomonas putida* is known to be resistant to high concentrations of rhamnolipids showing little change in growth rate if exposed to concentrations up to 90 g L^−1^ (Wittgens et al. 2011). The biomass concentration of 5.5 g L^−1^ and rhamnolipid productivity of 0.05 g g^−1^ glucose observed here are similar to previously reported yields (Beuker et al. 2016). Although a great deal of literature exists for the influence of nitrogen limitation on microbial carbohydrate formation, little is known about the role of glucose limitation on rhamnolipid production (Guerra-Santos et al. 1984). Here, glucose-limited feeding regimes were used and high titers of rhamnolipids were observed. Glucose limitation has also been reported to increase rates of other microbially produced surface-active products including more efficient production of surfactin with *B. subtilis* (Alonso and Martin 2016).

### Foaming of fermentation broth

#### Foam formation due to cells

Particle separation by foaming is well established through the technology of froth flotation. Particles with hydrophobic surfaces tend to attach to the foam air–water interface, whereas hydrophilic particles will not attach to it and remain in the bulk liquid (Alonso and Martin 2016). Rhamnolipids have been previously determined to be a dominant component responsible for intensive foaming during fermentative production (Long et al. 2016). Since the concentration of rhamnolipids in the fermentation broth was higher in the system without integrated adsorbent capture of rhamnolipids, it was expected that more intensive foam formation would occur in this reactor setup than when integrated adsorption was performed. Rhamnolipid enrichment in the foam phase was observed to be higher in the beginning of the fermentation at lower overall rhamnolipid concentrations, where presumably fewer molecules compete for the adsorption space at the bubble interface which allows formation of a lamellar rhamnolipid-layer. The foam that was formed was of a drier consistency due to enhanced drainage of production medium from the foam lamellae liquid space at this stage. A similar trend has been previously described when enrichment of 53-fold was achieved in a rhamnolipid producing system with *P. aeruginosa* (Heyd et al. 2011). It is known that the rhamnolipid-containing solutions can readily foam at the concentration of 19 mg L^−1^ rhamnolipids (Rashedi et al. 2006), so event at low concentration of rhamnolipids in the integrated system, foam was continuously produced until the time of 82 h. Structure and quality of the foam were changing over the fermentation time. The increase in the foam flow, presented in the Fig. 5, resulted in the apparent dilution of both the rhamnolipid and cell concentrations in the foam phase, therefore, the values of enrichment are below 1.

Interestingly, the foam flow rates measured here were the same in both fermentation setups. In the beginning of the foam formation, liquid flow in the foam fraction was lower than 10 mL min^−1^, which was concurrent with higher rhamnolipid enrichments in the foam. From the mid-point of fermentation time, high foam flow rates were observed with low rhamnolipid enrichments in both fermentation setups, although the rhamnolipid concentrations in the broth were not equal due to rhamnolipid accumulation in the non-integrated system and its separation in the integrated system. The foam flow rates did not seem to be influenced by the rhamnolipid concentration in the broth. Due to a linearly increasing foam flow rate over the course of fermentation, it could be suggested that foaming is indeed strongly influenced by increasing biomass concentration in the fermentation broth and not by the aeration rate, stirrer speed, pH and/or salt content, as these factors were kept identical across both fermentation processes. This effect has been previously reported (Sodagari and Ju 2014). It was observed that cells were the primary cause of broth foaming over the whole duration of the fermentation where a linear increase in foam flow and foam formation was observed in a *P. aeruginosa* fermentation process. It was also determined, that increasing rhamnolipid concentration resulted in decreased foam formation. This was likely due to increased viscosity of foams with higher rhamnolipid concentration and the formation of larger as well as different forms of micelles or physical aggregates that had lower foaming effects (Sodagari and Ju 2014). When foam fractionation was considered for both cell free and cell containing broths it was demonstrated that the presence of cells increased the foamability of the solution (Alonso and Martin 2016). Since foaming is an important pre-requisite for applying the foam adsorption technology it would be important to explain the reasons for this effect in rhamnolipid production systems. At such high flow rates, biomass and nutrient losses and their influence on system volumetric productivity cannot be ignored.

Future work will indeed be needed to investigate what effects the *P. putida* cells themselves or other biomolecules produced by the cells have on foam rates in fermentation cultures as foam formation did not seem to correlate with rhamnolipid enrichment here. Nevertheless, the foaming behavior permitted effective rhamnolipid capture using the integrated adsorption columns in this work.

### Rhamnolipid separation and recovery with integrated foam adsorption

#### Efficient separation of biosurfactant

During the fermentation, rhamnolipids accumulated from the liquid broth into the foam phase. An integrated adsorption column was used for rhamnolipid separation leveraging HHI between rhamnolipids and the adsorbent. This technique was found to be incredibly efficient and the cell-containing broth flow-through as well as the final culture broth in the reactor were found to have no detectable remaining rhamnolipids. This technology should be considered for application in the production of other biosurfactants, provided that foam fractionation of these amphiphilic compounds results in product accumulation in the foam. In systems where production efficiency is reduced by product inhibition or degradation, foam separation can be used to attain low, steady state, product concentrations in the bioreactor and separation of the product. For example, an increase in specific productivity by 30% was reported for nisin when feedback inhibition was reduced by foam fractionation and separation (Liu et al. 2010).

The elution method employed here allowed recovery of all of the rhamnolipids produced, even higher than previously reported recoveries (Beuker et al. 2016). Another important criterion for the comparison of product recovery efficiency is the product purity (Weber 2014), which is often not determined in the reported works. For each producing system with integrated foam fractionation and foam adsorption, a compromise must be made between product removal (recovery) and product enrichment (purity) by foam fractionation with the adjustment of parameters for an optimal foaming result. In the processes where the product is not inhibitory for the producing microorganism or degraded when excreted into the fermentation broth, product enrichment is the more important criterion, resulting in several benefits for the downstream process.

#### Influence of rhamnolipid enrichment on downstream processing

Since the foamate has a much smaller volume relative to the spent broth, foam fractionation results in a reduction of the liquid stream used for downstream processing (Stevenson and Li 2014). The efficiency of the adsorption process also depends on the concentration of product in the foam and its efficient adsorption to the adsorbate. Here, high rhamnolipid concentrations in the foam might have had a positive influence on adsorption capacity of the adsorbent. When high cell density cultivations are established, higher rhamnolipid production rates are expected. In this case it would be necessary to determine the optimal operating conditions, such as column size, number of columns and adsorption time, in order to not exceed the adsorption capacity limit for rhamnolipids.

Under traditional rhamnolipid production processes, these compounds are separated from the broth at the end of the fermentation process. Coupling rhamnolipid enriched foam to a packed adsorbent bed rather than solvent extraction from culture medium should result in reduced production costs due to lower handling volumes and, subsequently, lower amounts of solvents for the product recovery. An elution procedure using 3 BV of ethanol solution and 1 BV of methanol solution was used in this experiment. Complete rhamnolipid separation was possible, since no other hydrophobic by-products were present in the fermentation broth, or significantly interacted with the adsorbent bed. Here, a high purity of rhamnolipids, 96%, was achieved using this simple purification method. The use of an integrated rhamnolipid separation in the fermentation process removed the need for installation of other foam destroying or collapsing devices as well as the use of any cell retention methods. Therefore, the downstream process is simplified by integrated foam adsorption method presented here, and processing time is significantly reduced. The reduced number of unit operations and the high rhamnolipid purity in this system could significantly improve the economics of the rhamnolipid production by reducing the downstream processing costs. The proposed fermentation technology fully conforms to the principles of process intensification and could be readily scaled. Integrated foam adsorption exhibits the following improvements for rhamnolipid production by fermentation: (1) foam can be successfully collapsed by HHI on the adsorbent surface in the integrated adsorption column, (2) rhamnolipids are completely separated from the fermentation broth and bound to the adsorbent, and (3) biomass and broth recirculation promoted constant system productivity.

In this study, rhamnolipid biosurfactants were concentrated and recovered from cell-containing nutrient broth of *P. putida* EM383 using foam fractionation coupled to packed bed adsorption technique and biomass recirculation in batch mode. The results suggest that a simple foam adsorption method can be used for the concentration and primary purification of rhamnolipid biosurfactant products. The proposed production process led to high yield and purity of rhamnolipid from the microbial fermentation, is readily scalable, and will likely contribute to the economic feasibility of industrial-scale biosurfactant production. The method described here represents a promising technology for fermentative production and an effective separation of amphiphilic fermentation bio-products.

## Additional file

**Additional file 1: Figure S1.** Feeding rate applied during the fed-batch fermentation process is presented. Feeding pulse was performed every 30 min.

## Abbreviations

BV: bed volume
HHI: hydrophobic–hydrophobic interaction
ISPR: in situ product recovery
RL: rhamnolipid
