# Supplementary figures and images for: Production of rhamnolipids by integrated foam adsorption in a bioreactor system
Source: AMB Express. 2018 Jul 24;8:122. doi: 10.1186/s13568-018-0651-y (PMC6057861; doi:10.1186/s13568-018-0651-y)

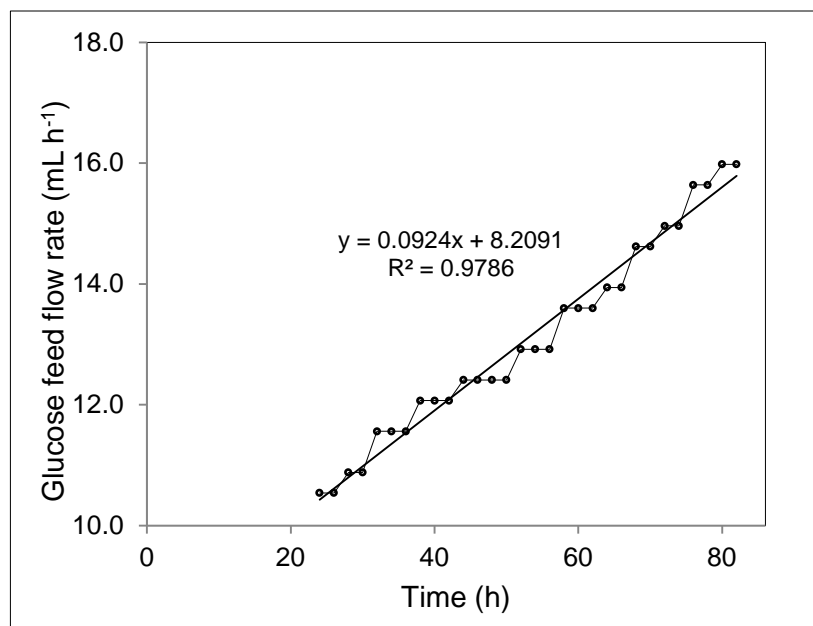

Supplement: Supplementary file 1 — Additional file 1: Figure S1. Feeding rate applied during the fed-batch fermentation process is presented. Feeding pulse was performed every 30 min. [file 13568_2018_651_MOESM1_ESM.pdf]
